# The Effect of Fineness on the Hydration Activity Index of Ground Granulated Blast Furnace Slag

**DOI:** 10.3390/ma12182984

**Published:** 2019-09-15

**Authors:** Jinpeng Dai, Qicai Wang, Chao Xie, Yanjin Xue, Yun Duan, Xiaoning Cui

**Affiliations:** 1 National and Provincial Joint Engineering Laboratory of Road & Bridge Disaster Prevention and Control, Lanzhou Jiaotong University, Lanzhou 730070, China; daijp@mail.lzjtu.cn (J.D.); bridgeconstruct@sina.com (Y.X.); 2 Civil Engineering Department, Lanzhou Jiaotong University, Lanzhou 730070, China; vipbridge@126.com (C.X.); concretebridge@sina.com (Y.D.); concretematerials@yeah.net (X.C.)

**Keywords:** ground granulated blast furnace slag, fineness, hydration activity index, gray correlation analysis

## Abstract

To improve the properties of ground granulated blast furnace slag (GGBS) and utilize ground granulated blast furnace slag efficiently, this study investigates the effect of fineness on the hydration activity index (HAI) of ground granulated blast furnace slag. The hydration activity index of GGBS with six specific surface areas (SSAs) was characterized by the ratio of compressive strength of the prismatic mortar test block. The particle size distribution of GGBS with different grinding times was tested by laser particle size analyzer. The paste of different specific surface area GGBSs in different curing ages was investigated at the micro level by X-ray diffraction, scanning electron microscope, energy dispersive spectrometer, thermogravimetric scanning calorimeter, and differential scanning calorimeter. The effect of particle distribution of GGBS on the hydration activity index of different curing ages was studied by gray correlation analysis. The results indicated that the compressive strength and hydration activity index increases with the increase of a specific surface area of GGBS at different curing ages. The hydration activity index at different curing ages is almost a linear role for specific surface areas. With the increase in the specific surface area of GGBS, the content of Ca(OH)_2_ in paste decreases gradually. When GGBS was added into a mortar test block, the hydrate calcium silicate gel in paste changed from a high Ca/Si ratio to a low Ca/Si ratio. The 0–10 micron particles of GGBS particle distribution were highly correlated with the hydration activity index at different curing ages.

## 1. Introduction

Slag is a granular substance of molten blast furnace slag quenched by water. Because minerals cannot crystallize in time, slag contains 80% to 90% glass phases, and it has certain potential activities. With the development of green high performance concrete, ground granulated blast furnace slag has obtained an important auxiliary cementations material of concrete, also known as the “sixth ingredient” of concrete. With the deepening of the research on ground granulated blast furnace slag, many of its excellent characteristics have been gradually discovered. Jaewon and Namgi [1] studied the effects of rapidly chilled and air-cooled nano slag as mineral admixtures on the properties of micro silica mortar. The study found that increasing the fineness reduces its fluidity, while having larger particles surrounded by smaller particles improves its strength. Sharmila and Dhinakaran [2,3] studied the effect of ground nano slag on the compressive strength, porosity, sorption, and resistance to chloride ion penetration of high strength concrete. They found that the strength and durability of high strength concrete were best when the ground nano slag content was 10%. In addition, a smaller ratio (5%) of the ground nano slag is not well dispersed, and is not enough to obtain strength. A large ratio (15%) of ground nano slags leads to an increase in the aggregated ultrafine particle size of concrete and the improper filling of pore. Susanto [4] has conducted experimental studies on the compressive strength, flexural strength, elastic modulus, chloride ion migration, and resistivity of concrete mixed with ultra-fine slag powder. It was found that concrete mixed with ultrafine slag powder has higher early strength, lower permeability and better durability at the curing age of 3 days. Quanlin and others [5] studied the hydration degree of ultrafine mineral powder with a specific surface area of 300 to 800 m^2^/kg to explain the compaction effect and pozzolanic effect of ultrafine mineral powder, and the results showed that the addition of ultrafine filler improved the porosity and pore diameter distribution of paste. Kefei and others [6] studied the pore structure of cement based materials with or without 70% ground slag, and the results showed that ground granulated blast furnace slag significantly refined the pores, significantly improved the specific surface area and fractal dimension, and moved the pore distribution from the range of 10 nm–100 nm to the range of <10 nm. Caijun et al. [7] studied the flow ability, compressive strength, heat of hydration, porosity and calcium hydroxide content of ultra-high-strength concrete (UHSC) with cement–silica fume–slag binder. It was found that slag reduced the flow ability, compressive strength, porosity, and calcium hydroxide content of UHSC to a certain extent. The ground granulated blast furnace slag was added to beam concrete and the stiffness of specimens with 50% and 70% content is 10% and 4% higher than that without ground granulated blast furnace slag, but the stiffness of specimens without ground granulated blast furnace slag is 16% higher than that of specimens with 90% content. In order to reduce carbon emissions, it is feasible to add 70% ground granulated blast furnace slag (GGBS) in concrete mixtures [8]. Adding superfine ground granulated blast furnace slag can make the self-compacting concrete reach a higher level of strength [9,10]. Calcium nitrate combined with ultrafine ground granulated blast furnace slag as an admixture to be added to concrete demonstrated that these admixtures are effective in improving the mechanical properties and corrosion resistance of concrete [11]. Hadjsadok et al. [12] used granulated blast furnace slag with low reactivity index to modify mortar and studied the microstructure and durability of the mixture. The results showed concrete with 30% substitution can obtain good mechanical properties and durability. The hydration and strength of cement are studied by using a grinder to activate the slag and clinker, respectively, and mechanical activated granular blast furnace slag was used to replace the clinker in Portland Slag Cement (PSC). The results show the change of microstructure caused by the increase of reactivity, and that densification in slag is related to the increase of cement strength. From the perspective of strength development, the fineness of slag is more critical than the fineness of a clinker [13]. The chemical composition, particle morphology, water demand, and hydration characteristics of different granulated blast furnace slag were investigated. The results showed that there was no significant difference in the chemical composition and particle morphology of GGBS with changes to the GGBS particle size, but also showed that the change rate of water demanded and the intensity changed rapidly with the change of GGBS particles [14]. Using ultrafine ground granulated blast furnace slag (UFS) as a substitute for cement accelerates the hydration of the admixture paste. The initial setting time of the paste depends not only on the dosage of UFS, but also on its fineness [15]. The mechanical properties and durability of concrete depends to a large extent on the hydration activity of the GGBS [16,17]. The activity and properties of slag powder mainly depend on the glass content, the chemical composition of slag and the fineness of slag powder. Since the glass content and chemical composition of slag are determined passively by the iron making process, the activation performance of slag powder can only be changed by changing the fineness of slag powder. The fineness of slag powder is usually expressed by a specific surface area and particle size distribution. Different levels of fineness can be achieved by grinding different materials with different grinding methods and different grinding times [18,19,20,21].

To further explore the effect of GGBS fineness on hydration activity, granulated blast furnace slag was pulverized by ball mill into six different specific surface area slag powders, and the influence of specific surface area on hydration index of ground granulated blast furnace slag at different ages was analyzed. The relationship between hydration activity indexes and the specific surface area of ground granulated blast furnace slag at different ages was established. The effect of a specific surface area of ground granulated blast furnace slag on the composition and microstructure of the paste was analyzed by using XRD, SEM, EDS, and TG-DSC at different curing ages. The effects of different particle size distributions of hydration activity index at different ages were analyzed by using gray system theory.

## 2. Materials and Methods

### 2.1. Material Properties

**Figure 1:**
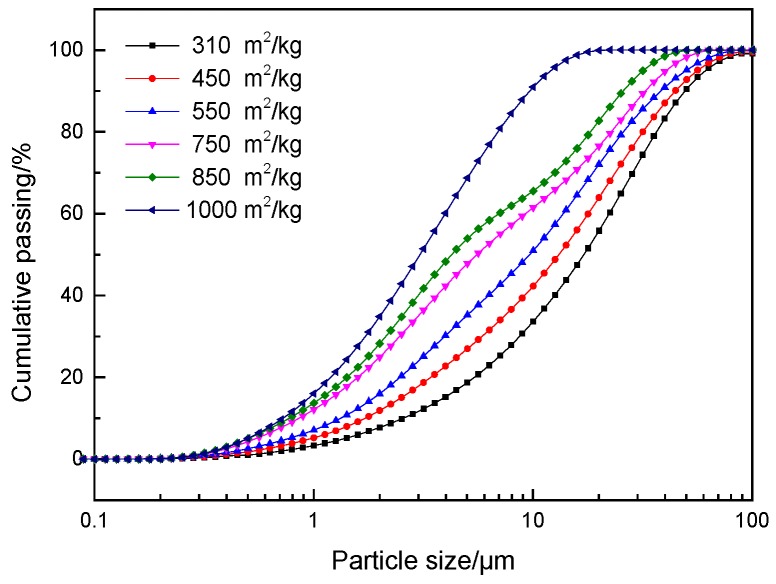
Particle size distribution curves of ground granulated blast furnace slag (GGBS).

**Figure 2:**
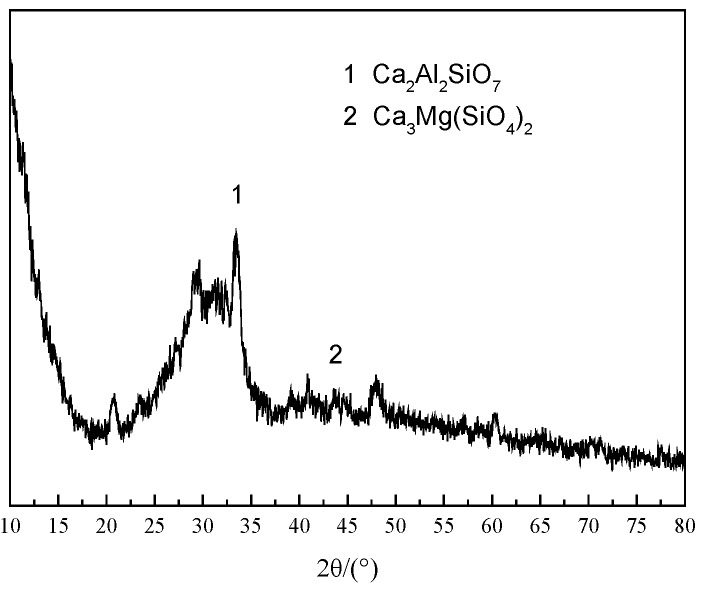
XRD patterns of GGBS.

Ordinary Portland cement (P.O 42.5) was provided by Qilian Mountain Cement Co., Ltd. (Lanzhou, China), the specific surface area of the cement was 326 m^2^/kg. The granulated blast furnace slag was provided by Jiuquan Iron and Steel Co. Ltd. (Jiayuguan, China), and was ground into slag powder with different specific surface areas in the ball mill. The specific surface areas were 310 m^2^/kg, 450 m^2^/kg, 550 m^2^/kg, 750 m^2^/kg, 850 m^2^/kg, and 1000 m^2^/kg. X-Ray Fluorescence Spectrometry (XRF) and Laser particle size analysis examinations were used to analyze the chemical composition and particle size distribution of materials. The Chemical compositions of cement and GGBS are given in Table 1. The main components of GGBS used in this study are CaO and SiO_2_, accounting for 72.3%, and the other main elements are Al_2_O_3_ and MgO, accounting for 7.22% and 7.32%, respectively. The particle size distribution of GGBS with different specific surface areas is shown in Figure 1. Figure 2 shows the XRD result for GGBS. There were a low number of peaks, only cacoclasite and merwinite, meaning no crystalline phases were detected in the GGBS. Having no crystalline phase and small particle size of GGBS indicates the mineral admixture has a high degree of reactivity [4]. The ISO standard sand was provided, while the quality assurance of sand was controlled as per the CNS (Chinese National Standard) GB/T 17671 [22].

### 2.2. Characterizations

#### 2.2.1. Compressive Strength

Mortar tests blocks were made according to CNS GB/T 17671 [23] and CNS GB/T18046 [24]. The hydration activity index is calculated by the ratio of the compressive strength of the test mortar and the compressive strength of reference mortar. The mortar mixes ratio is shown in Table 2. The water-binder ratio in this study is 0.5, while the GGBS content by weight is 50%. In this paper, the sample name Cement indicates that the cementitious material in the sample is cement only, while KA, KB, KC, KD, KE, and KF indicate that the cementitious material in the sample contains GGBS with specific surface areas of 310 m^2^/kg, 450 m^2^/kg, 550 m^2^/kg, 750 m^2^/kg, 850 m^2^/kg, and 1000 m^2^/kg, respectively.

Cement, water, and GGBS were weighed. Specimen were mixed using a paddle mixer and the following procedure. After thoroughly mixing the cement with GGBS, water was added to the mixture in a blender. The mixture was initially mixed at 140 revolutions per minute (rpm) for 30 s, then sand was added at the beginning of the second 30 s, and then the materials were mixed for 30 s at 285 rpm. While pausing for 90 s, the cement paste adhering to the sides of the mixing bowl was scraped in the first 15 s. The entire mixture was mixed for another 60 s at 285 rpm. After the test materials were mixed, the sample was loaded into a prismatic mound with a side length of 40 mm × 40 mm × 160 mm. Then the Mortar specimens were cured in mound for 24 h in the environment where the temperature was 20 ± 2 °C, and the relative humidity was not less than 95%. Afterwards, the specimens were demounted and maintained until the test age. Test the compressive strength of the test mortar and reference mortar at 1 day, 3 days, 7 days, 14 days and 28 days.

#### 2.2.2. Hydration Activity Index

To test the compressive strength of the specimen at different ages, the hydration activity indexes (*HAI*) of GGBS were calculated at different ages according to Formula (1).

(1) $$HAI,\%=\frac{f_{t}}{f_{0t}}\times 100$$

where: *HAI* is hydration activity index of GGBS (%); *f_t_* is compressive strength of test mortar at the *t* age (MPa); *f*_0*t*_ is the compressive strength of reference mortar at the *t* age (MPa).

#### 2.2.3. X-Ray Diffraction Analysis

X-ray diffraction (XRD) examination was used to analyze the phase composition of mortar specimens. After the compressive strength test, the block paste is taken from the compressive strength test block and placed in anhydrous ethanol to terminate the hydration [25]. After that, the block paste was dried and ground into powder, and the powder was used to prepare XRD analysis samples through the square mesh sieve with 75 microns. The XRD patterns of GGBS with different specific surface areas at 1 day, 3 days, 7 days and 28 days were tested.

#### 2.2.4. SEM Analysis

Scanning electron microscopic examination (SEM) was used to analyze the microstructure of mortar specimens. After the compressive strength test, the block paste is taken from the compressive strength test block and placed in anhydrous ethanol to terminate the hydration. After that, the block paste was taken out and dried to prepare SEM analysis samples.

#### 2.2.5. TG-DSC Analysis

The content of Ca(OH)_2_ in hydrate cement paste was determined by TG-DSC analysis. Using the sample obtained from Section 2.2.1, the bulk paste was put into anhydrous ethanol to terminate hydration, then it was dried and ground into powder. The powder was prepared using a 75 micron square-hole sieve, and 50 milligram samples were taken for test. Thermal analysis is performed between 20 and 1000 °C, in an N_2_ atmosphere (20 mL/min). The heating rate is 10 °C/min and an alumina crucible is used to keep the specimens. The Ca(OH)_2_ content was calculated based on the ignited weight of the sample.

### 2.3. Principle of Gray Relational Analysis

The principle of gray relational analysis is based on the geometric approximation of micro or macro factors in relation to each other [26]. It is an analysis to analyze and determine the degree of influence on factors or contribution of several sub-factors to main factors [27]. The detailed calculation formulas are as follows

(2) $$\begin{array}{c} \left\{X_{0}^{\left(0\right)}\left(i\right)\right\},i=1,2,\ldots ,N_{0}\\ \left\{X_{1}^{\left(0\right)}\left(i\right)\right\},i=1,2,\ldots ,N_{1}\\ \left\{X_{2}^{\left(0\right)}\left(i\right)\right\},i=1,2,\ldots ,N_{2}\\ \ldots \ldots \\ \left\{X_{k}^{\left(0\right)}\left(i\right)\right\},i=1,2,\ldots ,N_{k} \end{array}\}.$$

Among them, *N*_1_, *N*_2_, …, *N*_k_ belongs to the natural number set and is not necessarily equal. *k* represents *k* influencing factors. The array {$X_{0}^{\left(0\right)}$(*i*)} is assigned to main-array and {$X_{m}^{\left(0\right)}$(*i*)} (*m* = 1, 2…*k*) to sub-arrays.

(3) $$\bar{X_{m}}=\frac{1}{N_{m}}\sum_{i=1}^{N_{m}}X_{m}^{\left(0\right)}\left(i\right)$$

The conversion $Y_{m}\left(i\right)=X_{m}^{\left(0\right)}\left(i\right)/\bar{X_{m}}$ is made and then following arrays called inverted arrays can be obtained:

(4) $$\begin{array}{c} \left\{Y_{0}\left(i\right)\right\},i=1,2,\ldots N_{0}\\ \left\{Y_{1}\left(i\right)\right\},i=1,2,\ldots N_{1}\\ \left\{Y_{2}\left(i\right)\right\},i=1,2,\ldots N_{2}\\ \ldots \ldots \\ \left\{Y_{k}\left(i\right)\right\},i=1,2,\ldots N_{k} \end{array}\}$$

The absolute difference Δ*_om_*(*i*) between $\left\{Y_{m}\left(i\right)\right\}$ and $\left\{Y_{0}\left(i\right)\right\}$ at *t* = *i* is:

Δ*_om_*(*i*) = |*Y*_0_(*i*) − *Y_m_*(*i*)|, *i* = 1, 2, ... *k* (5)

Find the maximum values $\underset{i}{max}\underset{k}{max}\left|Y_{k}(i)-Y_{0}(i)\right|$ and minimum values $\underset{i}{min}\underset{k}{min}\left|Y_{k}(i)-Y_{0}(i)\right|$ in Δ*_om_*(*i*) nd mark them as *m* and *M*, calculate correlation coefficients of each comparison sequence and corresponding elements of reference sequence, as shown in Equation (6).

(6) $$r\left(y_{0}\left(i\right),y_{m}\left(i\right)\right)=\frac{m+\rho \times M}{\Delta_{k}\left(i\right)+\rho \times M}$$

In the formula, *ρ* is the resolution coefficient, which is used to weaken the influence of distortion of correlation coefficient. The coefficient is artificially introduced to improve the significance of the difference between correlation coefficients. the coefficient value is between 0 and 1, and the smaller *ρ* is, the greater difference there is between the correlation coefficients and the stronger distinguishing ability.

Because the degree of correlation between each comparison sequence and reference sequence is reflected by *N* correlation coefficients, the correlation information is scattered. For convenience of the overall comparison, correlation information is centralized. The average value is used to centralize the information, as shown in Equation (7).

(7) $$r_{i}=\frac{1}{N}\sum_{i=1}^{n}r(y_{0}(i),y_{m}(i))$$

Because $\left|Y_{0}\left(i\right)-Y_{m}\left(i\right)\right|$ is used in calculating Δ*_om_*(*i*) the polarity of correlation between factors cannot be distinguished. The formulas below are used to judge it:

(8) Qm=∑i=1NiYm(i)−∑i=1NYm(i)∑i=1NiNQi=∑i=1Ni2−(∑i=1Ni)2/N}

When sign $Q_{m}/Q_{i}$ = sign $Q_{0}/Q_{i}$,$Y_{m}$ and $Y_{0}$ are positively correlated. When sign $Q_{m}/Q_{i}$ = −sign ($Q_{0}/Q_{i}$), $Y_{m}$ and *Y*_0_ are negatively correlated.

The gray correlation degree is the quantitative value of the correlation degree among factors. The higher correlation degree, the stronger correlation between main-factor and sub-factor. The positive correlation between main-array and sub-array indicates that the sub-array can enhance the main-array. However, the negative correlation between main-array and sub-array indicates that the sub-array will weaken the main-array [27].

## 3. Test Results and Discussion

### 3.1. Compressive Strength Analysis

The compressive strength of the specimen at 1 day, 3 days, 7 days, 14 days and 28 days is presented in Table 3. It can be seen that the compressive strength of the specimens increases with the increasing of its age. The compressive strength of the reference mortar at the same age increases with the increase of the specific surface area of the GGBS. 

### 3.2. Hydration Activity Index Analysis

**Figure 3:**
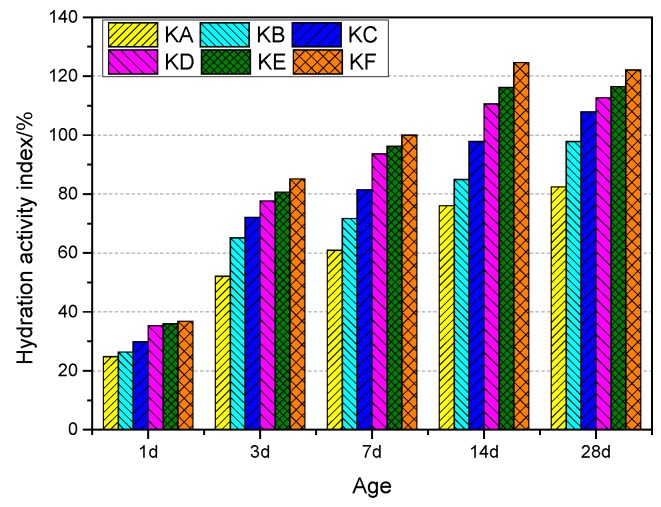
*HAI* of GGBS.

**Figure 4:**
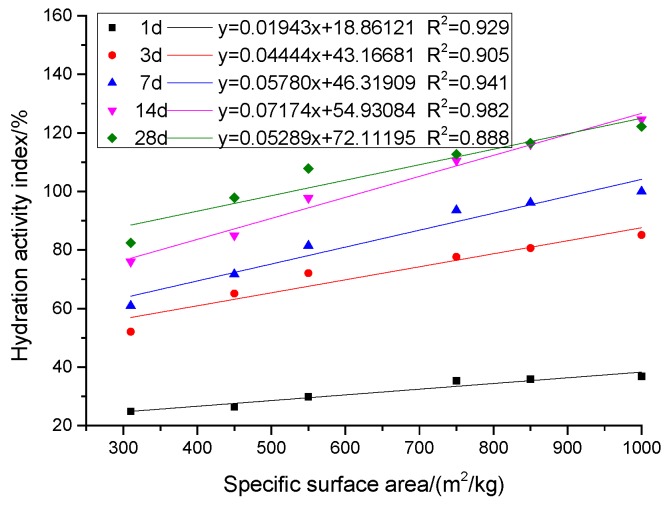
Relationship between *SSA* and *HAI* of GGBS.

The hydration activity indexes of GGBS with different specific surface areas at different ages are shown in Figure 3. The relationship between specific surface area of GGBS and hydration activity index at different ages is shown in Figure 4. At each age, the change of hydration activity index is basically the same, with the increase of specific surface area of GGBS, the hydration activity index increases gradually. On the 7th day, the hydration activity index of KA to KF was 60.94%, 71.72%, 81.48%, 93.60%, 96.16%, and 100.00%, respectively. The hydration activity index of KF was 1.64, 1.39, 1.23, 1.07, and 1.05 times KA to KE. At 28 days of age, the hydration activity index increased to 82.43%, 97.84%, 107.84%, 112.70%, 116.49%, and 122.16%, respectively. The hydration activity index of KF was 1.48, 1.25, 1.13, 1.08 and 1.05 times KA to KE. In addition to KF (1000 m^2^/kg), the hydration activity index of GGBS with a different specific surface area increased with age. The hydration activity index of the KF specific surface area increased with age before 14 days and decreased after 14 days. Figure 4 shows the relationship between the specific surface area and the hydration activity index of GGBS. It is observed that the hydration activity index is almost a linear function of the specific surface area at different ages. Linear regression was performed using the least squares method, and the regression coefficient R^2^ was greater than 0.888.

With the increasing specific surface area of GGBS, the proportion of fine particles of GGBS increases, leading to a more uniform and effective filling of the voids of cement particles. The larger the surface area, the stronger the reaction ability, while the faster the reaction rate, the more complete the reaction and the higher the strength of the test mortar. At the same time, the fine slag particles which are not involved in the hydration reaction are uniformly dispersed in the pores, while the gel body, which acts to fill the pores and pore cracks, improves the pore structure and improves the compactness of the cement stone. In addition, the fine mineral powder particles act as a skeleton of the micro-aggregate, so that the cementitious material has a better particle gradation, forming a self-tight packing system with a dense filling structure and a meso-level layer, The gel structure is further optimized, and the interfacial adhesion between the aggregates and the microstructure of the mortar is improved, thereby improving the macroscopic performance of the mortar [18,19].

The specific surface area of KF is 1000 m^2^/kg, the particles below 3.43 micron account for 50%, and the particles below 10.85 micron account for 90%. This will increase the chance of early contact with Ca(OH)_2_, which would increase the hydration speed and the number of available hydration products. At the later stage of hydration, the small and medium particles of KF are almost consumed, and the rate of hydration products is reduced. However, in the reference mortar, the cement is still hydrating to form hydration products to fill the voids. As a result, the hydration activity of KF decreases after 14 days.

### 3.3. X-Ray Diffraction Analysis

**Figure 5:**
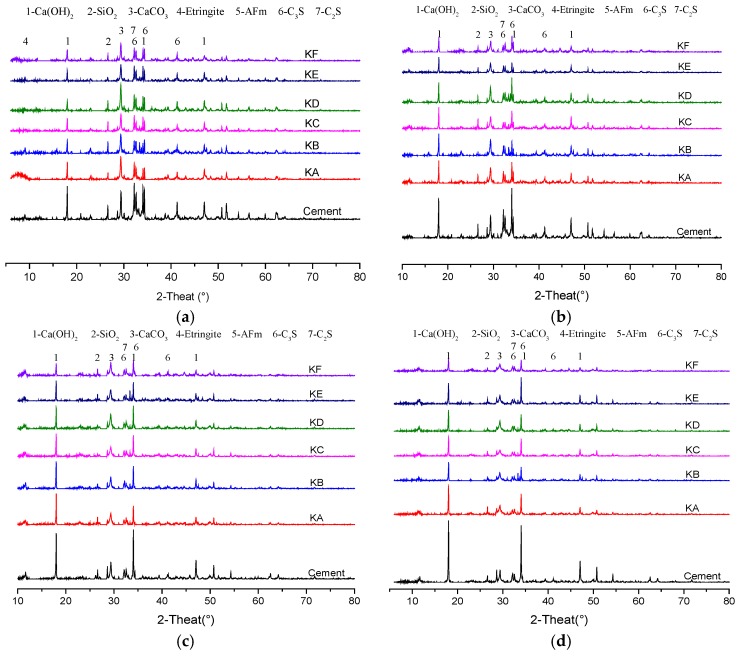
XRD patterns of specimens with different *SSA*. (**a**) XRD patterns of specimens at 1 day; (**b**) XRD patterns of specimens at 3 days; (**c**) XRD patterns of specimens at 7 days; (**d**) XRD patterns of specimens at 28 days.

Figure 5 shows the XRD patterns of granulated blast furnace slag at different specific surface areas for 1 days, 3 days, 7 days, and 28 days. The diffraction peaks of Ca(OH)_2_ are mainly at 18.048°, 34.110°, and 47.129°. After one day of hydration, the obvious diffraction peaks of Ca(OH)_2_ can be seen in the slurry hydration products. In addition, there are a few characteristic peaks of CaCO_3_ (peak 29.399°), which involve the carbonization products of Ca(OH)_2_ during the hydration process. Among the mineral composition of the cement clinker, the main XRD peaks of tricalcium silicate (C_3_S) are 32.192°, 34.377°, and 41.297°. The main XRD peaks of dicalcium silicate (C_2_S) are 32.136°, 32.469°, and the characteristic peak of tetracalcium ferrialuminate (C_4_AF) is weak. No obvious peak of tricalcium aluminate (C_3_A) was found in the XRD diffraction pattern because of its faster hydration rate. Compared with GGBS with a different specific surface area, the diffraction peak of Ca(OH)_2_ decreases with the increase in the specific surface area of granulated blast furnace slag. This indicates that granulated blast furnace slag participates in the hydration reaction from the beginning of hydration. The larger the specific surface area of granulated blast furnace slag, the more intense the reaction with Ca(OH)_2_ in a porous solution and the greater the consumption of Ca(OH)_2_ in the solution.

The hydration of Portland cement is represented by a series of chemical equations. Each equation defines the basic reaction of a different clinker. In the case of gypsum in the mixture, the following equations are generally proposed [28,29,30].

(9) $$C_{3}S+5.3H\to C_{1.7}SH_{4}+1.3CH$$

(10) $$C_{2}S+4.3H\to C_{1.7}SH_{4}+0.3CH$$

(11) $$\begin{array}{cc} C_{3}A+3C\overset{-}{S}H2+26H\to & C_{6}A{\overset{-}{S}}_{3}H_{32}\\ & \left(AFt\right) \end{array}$$

(12) $$\begin{array}{cc} C_{6}A{\overset{-}{S}}_{3}H_{32}+2C_{3}A+4H\to & 3C_{4}A\overset{-}{S}H_{12}\\ & \left(\mathrm{AFm}\right) \end{array}.$$

In the active mineral admixture, active SiO_2_ can react with calcium hydroxide and high-purity calcium silicate hydrate to form more stable calcium silicate hydrate. At the same time, active Al_2_O_3_ can react with calcium hydroxide to produce hydrate calcium aluminate. The main reactions are as follows [31].

(13) $$\begin{array}{cc} 3Ca{\left(OH\right)}_{2}+\mathrm{Slag}+H_{2}O\to & 3CaO\cdot 2SiO_{2}\cdot 3H_{2}O+4CaO\cdot {\mathrm{Al}}_{2}O_{3}\cdot 3H_{2}O\\ & \left(SecondaryC-S-H)(SecondaryC-A-H\right) \end{array}$$

The vitreous body of granulated blast furnace slag is destroyed in the grinding, which promotes the activities of CaO, SiO_2_, and Al_2_O_3_. These active CaO, SiO_2_ and Al_2_O_3_ can further react with Ca(OH)_2_ produced by hydration of C_3_S and C_2_S in cement to form calcium silicate hydrate and calcium aluminate hydrate, which further exerts the potential hydraulic property of slag. During the grinding, the particle size of slag powder decreases, while the crystal structure, chemical composition, and physical and chemical properties of slag itself change mechanically or chemically. The atomic structure of activated slag is rearranged and recrystallized, and the surface layer is reorganized spontaneously to form an amorphous structure. The irregularity of the internal crystal structure and transformation of multiphase crystal forms to make a lattice defect occur lead to an increase of the surface area and the surface energy. The mechanical properties, crystallographic properties, physical properties, and chemical properties of slag change regularly, which make the slag active. The smaller the specific surface area of GGBS, the finer the mineral powder, meaning the finer slag particles can quickly absorb the CH released by the cement hydration, and transform it into C-S-H gel and C-A-H with better strength and compactness. At the same time, because of the absorbing of CH, the hydration rate of C_3_S and C_2_S increased.

### 3.4. SEM-EDS Analysis

**Figure 6:**
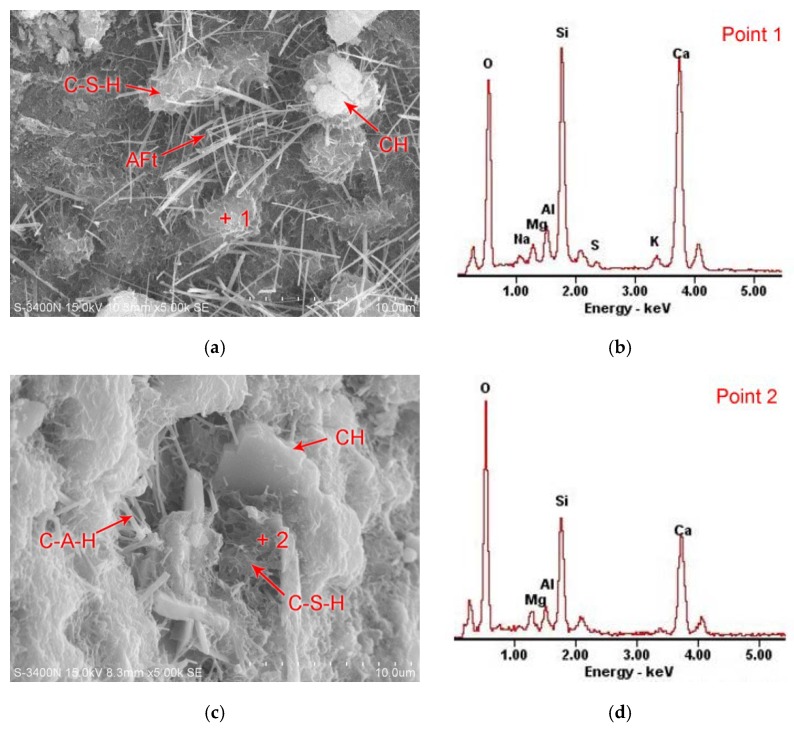

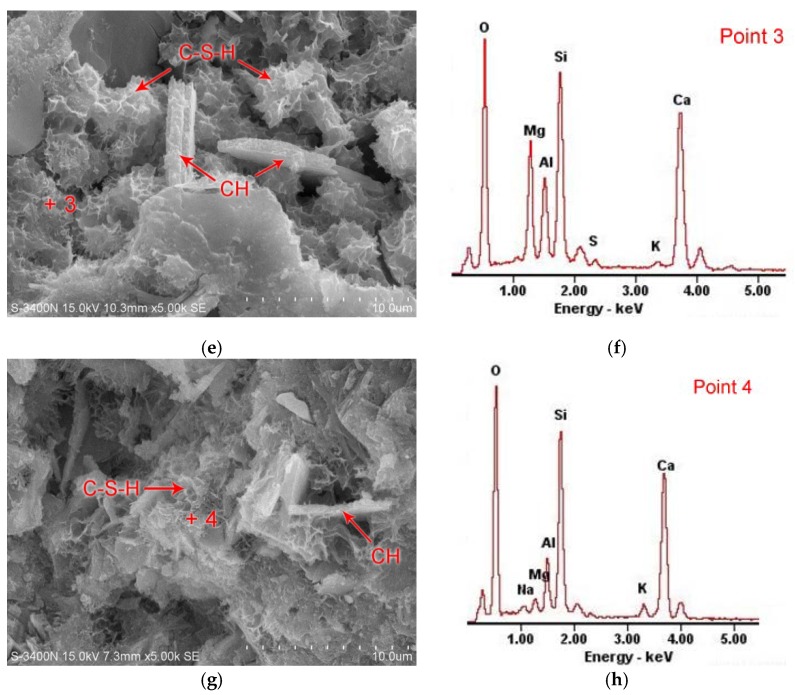
SEM micrographs of paste specimen of different *SSA* of GGBS and EDS in micro-area. (**a**) SEM micrograph of reference mortar at 28 days. (**b**) EDS of point 1 in micro-area of paste samples. (**c**) SEM micrograph of KB Test mortar at 28 days. (**d**) EDS of point 2 in micro-area of paste samples. (**e**) SEM micrograph of KD Test mortar at 28 days. (**f**) EDS of point 3 in micro-area of paste samples. (**g**) SEM micrograph of KF Test mortar at 28 days. (**h**) EDS of point 4 in micro-area of paste samples.

Figure 6 shows the SEM and EDS graphs of reference mortar, KB, KD, and KF at 28 days. As can be seen from Figure 6a, the reference mortar paste is mainly sheet-like CH and fibrous C-S-H, and a large number of needle-like Aft. From Figure 6c,e,g, it can be seen that the test mortar is mainly flaky CH and fibrous C-S-H. No obvious needle-shaped Aft can be seen, but rod-shaped C-A-H can be seen in the paste. From the analysis of EDS data in Figure 6b,d,f,h, it can be concluded that by adding GGBS with different specific surface area into the paste, the hydrate calcium silicate gel in the paste changes from a high calcium silicate ratio to a low calcium silicate ratio, so the composition of the cementitious material in the paste is improved.

The vitreous form of active SiO_2_ and Al_2_O_3_ in the ground slag powder is activated by mechanical grinding. Under the alkaline environment inside the concrete, it can react with the cement hydration product Ca(OH)_2_ to form a secondary hydration reaction, and produce hydrate calcium silicate and hydrate calcium aluminate with cementitious properties on the surface. Secondary hydration can promote further hydration of a cement clinker, resulting in a more stable and stronger low-alkalinity C-S-H gel. The enrichment of Ca(OH)_2_ in the interfacial transition zone is reduced, the orientation of Ca(OH)_2_ crystal in the interfacial transition zone is disturbed, and the mechanical properties of concrete are improved [32].

### 3.5. TG-DSC Analysis

**Figure 7:**
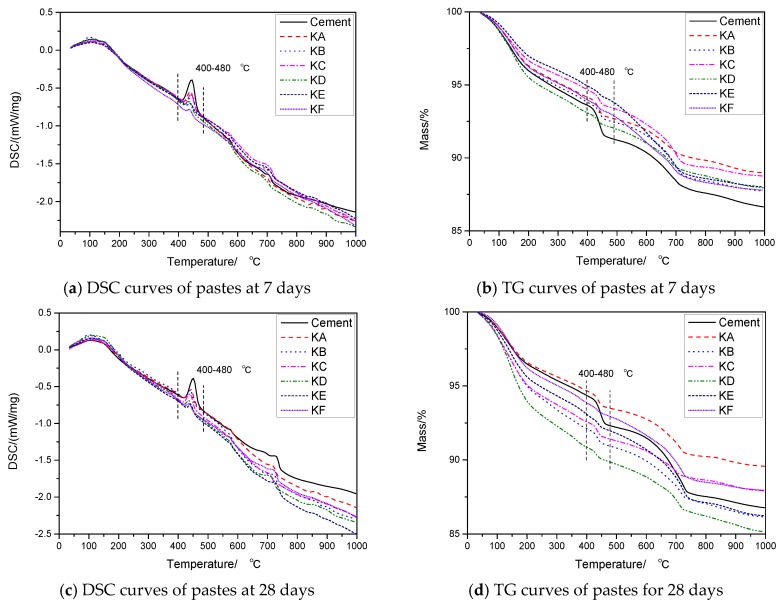
TG-DSC curves of pastes at different ages.

Figure 7 illustrates TG-DSC curves for test mortar and reference mortar pastes at various curing ages. It is evidently observed that every sample exhibited three obvious end thermal peaks at temperatures of around 120 °C, 450 °C, and 700 °C. It was reported the mass loss at three peaks range correspond to decomposition of hydrate (C-S-H, AFt), Ca(OH)_2_, and CaCO_3_, respectively [33,34,35]. This paper mainly analyzes the change of Ca(OH)_2_ content in the paste, as the deshydroxylation of Ca(OH)_2_ takes place between 400 and 480 °C. It can be seen from the figure that with the adding of GGBS, Ca(OH)_2_ in pastes decreases obviously. With the increase of specific surface area of GGBS, the content of Ca(OH)_2_ in paste decreases gradually at 7 days and 28 days, which is consistent with the result of XRD analysis above. Table 4 shows the content of Ca(OH)_2_ in pastes at 7 days and 28 days calculated by the method provided in reference [28]. It can be seen that the addition of GBBS at 7 days can reduce Ca(OH)_2_ by at least 42% in pastes and at by least 46% at 28 days.

### 3.6. Gray Correlation Analysis

Taking the hydration activity index of mortar at 1, 3, 7, 14, 28 days as the main-array (expressed as Y01, Y02, etc.) and the particle size distribution of GGBS with different specific surface area as the sub-array (expressed as Y1, Y2, etc.), the correlation between the sub- array and the main-array was calculated. The main-array and sub-array is shown in Table 5 and Table 6 respectively. The results of gray correlation analysis between particle size distribution and the hydration activity index of GGBS with different specific surface areas are given in Table 7.

It can be seen from Table 6 that the 0–5 micron and 5–10 micron particles are highly correlated with the GGBS hydration activity index. The mass fraction of particles at 0–5 micron has a maximum positive effect on its activity index at early age while particles at 5–10 micron contribute most at the later stage. The relative polarity of particles of particle size >10 micron in GGBS are negative, indicating that their anti-pressure activity is unfavorable.

## 4. Conclusions

The following conclusions were obtained from the experimental results:

(1) The compressive strength of the specimens increases with the increase of age. The compressive strength of the reference mortar at the same age increases with the increase of the specific surface area of GGBS.
(2) The hydration activity index of GGBS increased with the increase of the specific surface area. The hydration activity index of GGBS with different specific surface areas increased with the increase of age except in the case of KF. The hydration activity index of KF increased with the increase of age before 14 days and decreased after 14 days. The hydration activity index is almost a linear role of specific surface area at different curing ages.
(3) With the increase of the specific surface area of granulated blast furnace slag, the diffraction peak of Ca(OH)_2_ decreases gradually. With the adding of GGBS, the content of Ca(OH)_2_ in paste decreases obviously, and with the increase of specific surface area of GGBS, the content of Ca(OH)_2_ in paste decreases gradually.
(4) The hydrate calcium silicate gel in the paste is converted from a high Ca/Si ratio to low Ca/Si ratio by adding GGBS with different specific surface areas in the paste.
(5) 0–5 micron and 5–10 micron particles are highly correlated with the GGBS hydration activity index. The mass fraction of particles ranging 0–5 micron has a maximum positive effect on its activity index after a small number of days, while particles ranging 5–10 micron contribute most later on. The relative polarity of particles of particle size >10 micron in GGBS is negative.

## Figures and Tables

**Table 1:** Chemical composition of cement and GGBS/mass, %.

Chemical Composition/%	CaO	SiO_2_	Al_2_O_3_	Fe_2_O_3_	MgO	SO_3_	K_2_O	TiO_2_	ZnO
Cement	76.70	9.90	2.01	7.01	0.31	1.61	1.06	0.57	0.11
GGBS	42.90	29.40	7.22	2.26	7.32	2.86	1.10	0.81	-

**Table 2:** Mortar mixes ratio.

Raw Materials	Reference Mortar	Test Mortar
OPC/g	450 ± 2	225 ± 1
Mineral admixture/g	-	225 ± 1
ISO sand/g	1350 ± 5	1350 ± 5
Water/mL	225	

**Table 3:** Compressive strength of specimen, MPa.

Age	Cement	KA	KB	KC	KD	KE	KF
1 d	23.32	5.80	6.15	6.96	8.24	8.38	8.58
3 d	24.94	12.99	16.24	17.98	19.37	20.11	21.23
7 d	34.45	21.00	24.71	28.07	32.25	33.13	34.45
14 d	36.31	27.61	30.86	35.50	40.14	42.18	45.24
28 d	42.92	35.38	41.99	46.28	48.37	50.00	52.43

**Table 4:** Ca(OH)_2_ content of pastes at different ages,%.

Specimen	Age	
	7 Days	28 Days
Cement	9.50	8.68
KA	5.51	4.65
KB	4.73	4.48
KC	4.53	4.32
KD	3.66	3.29
KE	3.46	3.09
KF	2.72	2.51

**Table 5:** Time ranges of the main-array.

Specimen	Time-Array				
	*Y*_01_ (1 Day)	*Y*_02_ (3 Days)	*Y*_03_ (7 Days)	*Y*_04_ (14 Days)	*Y*_05_ (28 Days)
KA	0.789	0.722	0.726	0.748	0.773
KB	0.836	0.903	0.854	0.836	0.918
KC	0.947	1.000	0.970	0.961	1.012
KD	1.120	1.077	1.115	1.087	1.057
KE	1.139	1.118	1.145	1.142	1.093
KF	1.168	1.180	1.191	1.225	1.146

**Table 6:** Time ranges of sub-array.

Specimen	Time-Array					
	*Y*_1_ (0–5 μm)	*Y*_2_ (5–10 μm)	*Y*_3_ (10–20 μm)	*Y*_4_ (20–40 μm)	*Y*_5_ (40–60 μm)	*Y*_6_ (>60 μm)
KA	0.447	0.957	1.255	1.590	2.012	2.459
KB	0.644	0.983	1.224	1.340	1.594	1.915
KC	0.841	1.009	1.194	1.090	1.175	1.370
KD	1.141	0.880	0.848	1.057	0.920	0.256
KE	1.287	0.745	0.971	0.915	0.299	0.000
KF	1.640	1.425	0.508	0.006	0.000	0.000

**Table 7:** Gray correlation degree.

Age	Range of Particle Size/μm					
	0–5	5–10	10–20	20–40	40–60	>60
1d	+0.832	+0.825	−0.724	−0.704	−0.587	−0.472
3d	+0.809	+0.840	−0.738	−0.722	−0.603	−0.484
7d	+0.845	+0.837	−0.734	−0.716	−0.600	−0.485
14d	+0.846	+0.845	−0.735	−0.719	−0.601	−0.483
28d	+0.777	+0.838	−0.739	−0.723	−0.599	−0.477
